# Using administrative registries as a source for population-based cancer incidence and mortality

**DOI:** 10.1186/s12885-023-11754-w

**Published:** 2024-02-19

**Authors:** C. Corredor, M. Piñeros, C. Wiesner, E. de Vries

**Affiliations:** 1 Cancer Surveillance Group, National Cancer Institute, Bogotá, D. C Colombia; 2 Cancer Surveillance Section, International Agency for Research on Cancer, Lyon, France; 3 General Direction, National Cancer Institute, Bogotá, D. C Colombia; 4 Department of Clinical Epidemiology and Biostatistics. Faculty of Medicine, Pontificia Universidad Javeriana, Bogotá, D. C Colombia

**Letter to the Editor.**

**BMC CANCER.**

Dear Editor,

With interest we read the paper by Hernández et al. entitled “Patterns of breast, prostate and cervical cancer incidence and mortality in Colombia: an administrative registry data analysis” and recently published in BMC Cancer [1], based on data from the nation-wide operating Cuenta de Alto Costo (CAC). We feel obliged to express our concerns regarding these data. In the publication, the data referred to as *national cancer incidence and mortality* are not only far below the estimated incidence and mortality rates of the IARC–led Globocan 2020 project [2], but the incidence rates are much below those observed in the various high-quality Population-Based Cancer Registries (PBCR) existing in Colombia [3, 4]. The mortality numbers and rates are also much lower compared to the observed mortality as presented by the National Statistics Department (DANE) [5].

In the methods section, the authors chose to age-standardize rates using a very unconventional standard population (Latin American population estimated by the United Nations for 2019), which inhibits direct comparison of age-standardized rates (ASR), as no other cancer sources use this standard population. In addition, the authors also used the Colombian population estimated by DANE with cut-off date on June 30th, 2018 for comparisons of data between departments. As such, the resulting ASRs presented in the paper cannot even be compared between each other. The rationale behind using two distinct standard populations in the same paper, which moreover are uncommon to present cancer ASRs is unclear, furthermore hampering interpretation and comparison of the data. However, considering that the age-specific weights in LAC population estimated by the United Nations for 2019, are similar to the commonly used SEGI world standard population for the ages where cancer is common (middle age and onward), the resulting ASRs should be relatively similar and the observed differences will not be heavily influenced by the differences in standard population.

Table 1 presents the reported incidence from the four PBCR in Colombia which published their data in Cancer Incidence in 5 Continents volume XI (indicating good indicators of data comparability and completeness), as well as the data published by CAC. Comparing the observed incidence rates of breast, cervix uteri and prostate cancer reported for the period 2017 (average of the years 2015–2017) from these PBCR [3] with those reported by the CAC, it is clear that the rates provided by the CAC suffer from a very serious underestimation of around 50%. Breast and prostate cancer have shown an upwards trend in incidence in Colombia [3], so expected rates for 2018 in these registries would be expected to be even higher compared to those mentioned in Table 1. The breast cancer incidence rate (ASR) in the Colombian PBCRs is almost doubled compared to the rate presented by CAC and this relative difference is even bigger for cervix and prostate cancer.

**Table 1:** Comparison of reported incidence rates by the Cuenta de Alto Costo versus Population-Based Cancer Registries

Cancer type	Reports CAC (2018) [1]		Reported incidence rates from Population-Based Cancer Registries [3]							
			Cali (2017)		Bucaramanga (2017)		Manizales (2017)		Pasto (2017)	
	**N**	**ASR**^**a**^	**CR**	**ASR**^**b**^	**CR**	**ASR**^**b**^	**CR**	**ASR**^**b**^	**CR**	**ASR**^**b**^
**Breast**	4506	18.69	70.1	53.1	58.1	45.9	61.0	40.8	41.1	32.8
**Cervix uteri**	1425	5.93	18.7	14.7	14.3	11.3	16.5	11.5	22.1	17.1
**Prostate**	2593	11.34	66.8	55.4	44.6	40.6	67.4	45.4	42.6	35.1

^a^ ASR using the LAC population estimated by the United Nations for 2019; ^b^ASR using the SEGI world standard population

With respect to mortality, DANE is the National Colombian official source as well as the WHO mortality statistics databases. The CAC relies, according to the publication, on cancer mortality reported by the health insurers [1]. Table 2 clearly shows that relying solely on these reports underestimates site-specific cancer mortality. Differences in mortality were smaller than those observed for incidence, nevertheless, the lower rates reported by the CAC most likely indicate underreporting, especially for cervical and prostate cancer – again, ASRs must be compared with caution as different standard populations were used.

**Table 2:** Cancer (national) mortality 2018 according to DANE and Cuenta de Alto Costo

Source	Breast			Cervix uteri			Prostate		
	Deaths	CR	ASR^a^	Deaths	CR	ASR^b^	Deaths	CR	ASR^b^
**Infocancer – DANE** [5]	3441	13.8	11.0	1946	8.1	6.6	3182	13.6	10.2
**Cuenta de Alto Costo – CAC** [1]	2454	–	10.5	1021	–	4.3	1641	–	7.6

^a^ASR using the LAC population estimated by the United Nations for 2019; ^b^ASR using the SEGI world standard population; DANE: Departamento Administrativo Nacional de Estadística

The observed discrepancies most probably obey to the passive data collection method used by the CAC, which is highly prone to underreporting of cases by the health insurers and in turn, of their primary sources. This contrasts with active case finding following protocols by PBCR personnel [6–8], spending great efforts in exhaustive active case-finding rather than relying on passive reporting by third parties [6, 7]. According to the authors, “if there is any underreporting by the insurers this must be minimal because reporting is mandatory”. However, as has been also shown in communicable diseases, mandatory disease reporting by itself, is by no means a guarantee of complete reporting, and completeness is perhaps the most important quality indicator of a PBCR [6]. Such underreporting is likely, as not all health insurers have a good picture of their affiliated population at a given moment. In addition, the potential “sanctions” for health insurers which do not show good indicators of risk management, reflected by high proportions of late diagnoses, long waiting times for treatment, could contribute to underreporting of cases. Other potential explanations for the large divergence in incidence rates, such as differences between the mainly urban populations covered by the PBCR and the rest of the country, would not be able to explain such large differences. For cervical cancer in particular, rural and poor areas, not represented in the PBCR estimates, are known to be higher than those in the cities, so the difference between the reported and real, unobserved incidence, may be even larger.

The comparison combined with knowledge on cancer incidence and PBCR functioning, indicates a lack of completeness in the CAC data which has been repeatedly documented [7, 9]. The data presented in the paper do not support the statement of a very high completeness in the CAC [1]; the discrepancies observed should motivate a data linkage study between CAC and selected registries to establish potential failures to identify cancer cases and complete missing information on both sides.

Should the data of the CAC represent the “real” cancer incidence and mortality in Colombia, the incidence rates would be exceptionally low and would merit investigations to clarify the much lower than expected incidence of breast, cervical and prostate cancer. In addition, the Incidence: Mortality ratios would be exceptionally high, being inconsistent with previously reported survival rates of cancer in the country [10].

The data collected by the CAC are an important data source and of enormous potential to evaluate quality of oncological care in many aspects in addition to being a critical information source for population-based registries where they operate in the country. However, with the current indications for underreporting, relying on CAC incidence for planning and evaluation could have critical consequences. The much lower reported cancer rates informed in the paper by Hernández et al., may cause difficulties in resource allocation for cancer care, training of medical staff and research as the burden erroneously seems substantially lower than more reliable data from local, sub-national PBCR. When reporting “population-based” incidence and mortality rates it is always important to evaluate the important components of population-based cancer registry data, completeness and comparability being very vital there [11, 12].

It is important that CAC, PBCR and governmental institutions work together towards a collaboration which can help advancing to clearly depict the burden and epidemiological patterns of cancer in Colombia. A collaborative network integrating cancer information systems with the possibility to link individual data would provide a wealth of information which would benefit patients, healthcare professionals, decision makers and insurers alike.

## Data Availability

Not applicable, all cited numbers and figures have their respective bibliographic references.
